# QRNAS: software tool for refinement of nucleic acid structures

**DOI:** 10.1186/s12900-019-0103-1

**Published:** 2019-03-21

**Authors:** Juliusz Stasiewicz, Sunandan Mukherjee, Chandran Nithin, Janusz M. Bujnicki

**Affiliations:** 1 Laboratory of Bioinformatics and Protein Engineering, International Institute of Molecular and Cell Biology, 02-109 Warsaw, Poland; 2 Institute of Molecular Biology and Biotechnology, Faculty of Biology, Adam Mickiewicz University, 61-614 Poznań, Poland

**Keywords:** RNA, DNA, 3D structure, Molecular modeling, Structure refinement, AMBER force field, Software

## Abstract

**Background:**

Computational models of RNA 3D structure often present various inaccuracies caused by simplifications used in structure prediction methods, such as template-based modeling or coarse-grained simulations. To obtain a high-quality model, the preliminary RNA structural model needs to be refined, taking into account atomic interactions. The goal of the refinement is not only to improve the local quality of the model but to bring it globally closer to the true structure.

**Results:**

We present QRNAS, a software tool for fine-grained refinement of nucleic acid structures, which is an extension of the AMBER simulation method with additional restraints. QRNAS is capable of handling RNA, DNA, chimeras, and hybrids thereof, and enables modeling of nucleic acids containing modified residues.

**Conclusions:**

We demonstrate the ability of QRNAS to improve the quality of models generated with different methods. QRNAS was able to improve MolProbity scores of NMR structures, as well as of computational models generated in the course of the RNA-Puzzles experiment. The overall geometry improvement may be associated with increased model accuracy, especially on the level of correctly modeled base-pairs, but the systematic improvement of root mean square deviation to the reference structure should not be expected. The method has been integrated into a computational modeling workflow, enabling improved RNA 3D structure prediction.

## Background

Ribonucleic acid (RNA) molecules play pivotal roles in living organisms. RNAs are involved in a variety of biological processes: they transmit genetic information, they sense and communicate responses to cellular signals, and even catalyze chemical reactions [1]. With the very rapid discovery of new classes of RNA molecules, new functions beyond storing genetic information are also being discovered. The functions of RNA molecules and interactions of proteins, RNAs, and their complexes, often depend on their structure, which in turn is encoded in the linear sequence of ribonucleotide residues. Thus, the understanding of the molecular basis of RNA function requires the knowledge of RNA structure.

The experimental determination of RNA 3D structures is expensive and difficult [2, 3]. However, the ribonucleotide sequence determines RNA structure (in a similar manner as amino acid sequence determined protein structure), it is theoretically possible to infer the RNA structures from sequences. Since the historically first prediction of tRNA 3D structure in 1969 [4], throughout the decades, numerous computational methods were developed to generate RNA 3D structure from sequence. Currently, the field of research on RNA structure prediction is quite advanced, and the advantages and limitations of different methods are known, in particular from the assessment within the RNA-Puzzles community-wide experiment [5–7], which has been inspired by the CASP experiment for protein structure prediction [8].

Because of the very high costs of all-atom simulations, RNA 3D structures are usually not predicted by simulating all the details of the physical process of macromolecular folding, starting from sequence alone. The most successful general strategy for RNA 3D structure prediction that emerged from the RNA-Puzzles experiment involves the following approaches or their combination: 1) identification of pre-existing information in databases of molecular structure and e.g., using known structures as templates to develop a comparative model for the whole structure or its part; 2) running a simulation, often using a coarse-grained strategy, with restraints to represent all possible knowledge about the target structure, to generate ensembles of structurally similar conformations with possibly best scores. In this strategy, a typical approach is to derive potentials (scoring functions) based on a statistical analysis of experimentally determined structures. Statistical potentials can be used to replace or supplement the calculation of the physical free energy by evaluating the relative frequencies of features, such as pairwise distances of atoms (bonded and non-bonded) and mutual orientations of chemical groups (e.g., torsion angles). In this methodological framework, the most frequently observed structural features are also the most probable ones.

Simplifications applied in the process of RNA 3D structure prediction come with a cost of the loss of fine structural details. Computational models often present imperfect stereochemistry, unnatural bond lengths or steric conflicts. These deficiencies are clearly visible when using quality assessment tools, such as MolProbity [9, 10]. To obtain a high-quality model, a structure obtained from template-based modeling or from coarse-grained simulations needs to be further refined. However, even models perceived as correct by validation tools can still be far from their native structures. The most challenging task faced by the refinement is not only to improve the visible quality of the model but to bring it closer to the ‘true’ structure (which in case of real predictions is unknown at the time of the modeling). According to RNA-Puzzles, the best models of medium-sized RNA molecules exhibit root mean square deviation (RMSD) of 5–10 Å from the reference structure. It is tempting to ask whether a dedicated software tool could improve these results.

In this article, we present QRNAS, a new software tool for fine-grained refinement of nucleic acid structures, dedicated to improving the quality of models generated by low- to medium-resolution methods commonly used, e.g., for RNA 3D structure modeling. QRNAS is capable of handling RNA, DNA or chimeras and hybrids thereof, and enables modeling of nucleic acids containing modified residues. We demonstrate the ability of QRNAS to improve the quality of models generated in the course of RNA-Puzzles, often with improvement in the model accuracy, as compared to the reference structure. QRNAS is also able to improve MolProbity scores of NMR structures from Protein Data Bank.

## Implementation

### Force field

The force field used by QRNAS is a modified version of AMBER [11, 12] adopted to represent 107 modified nucleotides currently known to be present in RNA [13]. Currently, 130 residues are parametrized, including four canonical ribonucleotides (A, G, C, U) and deoxyribonucleotides (dA, dC, dG, dT) as well as naturally occurring modifications thereof (e.g., m7G, m1A, dU, wybutosine, queuosine, etc.). The key novel feature of QRNAS is an extension of the AMBER force field with energy terms that allow for modeling of restrained structures and enforce the backbone regularization. Imposition of secondary structure is also possible due to interaction types that go beyond the original AMBER force field, namely: explicit hydrogen bonds and enforcement of base pair co-planarity. These two interaction types are often poorly modeled in structures generated by computational structure prediction methods, and in our experience, their enforcement is a critical element of high-resolution refinement. Application of custom distance restraints required the introduction of pairwise harmonic interactions. Regularization of backbone torsions was realized by introduction of a knowledge-based energy term. All these add-ons carry along a certain degree of arbitrariness, and for this reason, we made them optional. In particular, our program falls back to plain AMBER [13] when all four additional terms are disabled. Similarly, electrostatics and van der Waals interactions can be disabled by the user (e.g., to speed up the calculation). With electrostatics enabled, the user can choose between generalized Born solvent and vacuum environment. In either case, the system is assumed to be non-periodic.

The new energy terms associated with hydrogen bonds, base pairs, backbone irregularities, and custom restraints are given, respectively, by Eqs. (1)–(4) (see below).

### Explicit hydrogen bonds

Although hydrogen bonds in AMBER are currently handled by means of electrostatic and van der Waals interactions, we decided to reintroduce an additional explicit description. Our goal was to gain finer control over the strength of this interaction. This was prompted in part by our observation, e.g., in the context of the RNA-Puzzles experiment, that in computational models of RNA structure obtained by low- to medium-resolution computational methods, interactions based on hydrogen bonding are often poorly modeled [5–7]. Computationally modeled structures often present an “almost correct” orientation of hydrogen bond donors and acceptors, which nonetheless deviates from the values typically observed in high-resolution structures. In these computational models, a relatively small adjustment of geometry often leads not only to an interaction that can be detected as a “proper” hydrogen bond by software for structure analysis but to an improved overall orientation of base moieties involved in pairing via these hydrogen bonds. Thus, with high force constant, explicit hydrogen bonds can be used as restraints when imposing secondary structure on the modeled nucleic acid molecule. Another benefit of enforcing strong hydrogen bonds in the structure optimization procedure is that geometrically correct contacts are preserved throughout the computational simulation once they are formed.

According to Lu et al., the statistical analysis of the hydrogen-bonds obtained from simulations shows that the strengths of hydrogen bonds in liquid water conform to a Gaussian distribution [14]. Therefore, the energy term associated with hydrogen bond (E_H-bond_) was chosen to be Gaussian in its length with an exponential dependence on the cosine of its angle:

1 $$ {E}_{H- bond}={k}_1\mathit{\exp}\left(-{r}_{ij}^2/d\right)\mathit{\exp}\left(\mathit{\cos}\left({\theta}_{ij k}-{\theta}_0\right)\right) $$

Where *k*_*1*_ denotes the force constant, *r*_*ij*_ is the hydrogen bond length between donor hydrogen *i* and acceptor *j*, and *θijk* is the bond angle between donor-hydrogen-acceptor. The parameters *k*_*1*_, *i*, *θ*_*0*_ were iteratively tuned to reproduce experimental hydrogen bond lengths. The multiplier was arbitrarily set at a value of − 1 kcal/mol, which proved to provide good persistence of contacts in the course of energy minimization.

### Base pair co-planarity

Models of RNA structure obtained by computational methods (in particular by coarse-grained methods and in the process of comparative modeling) often present various deviations of base-pair geometry. In particular, canonical Watson-Crick base pairs often deviate from co-planarity. Therefore, QRNAS was equipped with an optional feature that performs the idealization of base pair planarity. When enabled, Watson-Crick base pairs are not only restrained by explicit hydrogen bonds but also additionally flattened. The flattening is implemented by application of force to the atoms of each base according to Eq. (2):

2 $$ {E}_{BP}={k}_2{\sum}_{i\in base}{r}_{i0}^2 $$

where *k*_*2*_ denotes the force constant; *r*_*i0*_ is the distance from the *i*-th atom of the base to the plane that best matches the base pair. The plane is least-squares fitted to the atoms of both bases. The magnitude of the force acting on each atom is proportional to its distance from the plane of the base, while the direction of the force is perpendicular to this plane. Base pair restraints are introduced only at startup. For two Watson-Crick bases to be considered as a pair, the energy resulting from term (2) must be below − 2 kcal/mol. A user can also override this behavior by providing secondary structure in Vienna format (for a single chain) or as a list of contacts (in general case). In such case automatic detection of base pairs is disabled.

### Backbone regularization

The feature of backbone regularization is intended to correct outlying conformers reported by MolProbity. Upon energy minimization, it drags the backbone atoms of each residue to a known conformation, stored in an internal database. The database of preferred conformations was populated with data from all crystal structures of RNA stored in Protein Data Bank (PDB) [15] with a resolution below 1.4 Å as of June 2013. QRNAS identifies a local backbone conformation in a fragment stored in the database that is closest to the one in the input model according to a minimal Root Mean Square Deviation (RMSD) value. The forces acting on atoms are harmonic, as given by Eq. (3).

3 $$ {E}_{regul}={k}_3{\sum}_{i\in backbone}{\left(\overrightarrow{r_i}-\overrightarrow{b_i}\right)}^2 $$

The parameter *k*_*3*_ denotes the force constant; bi is the position of *i*-th backbone atom in a reference backbone. Coordinates *b*_*i*_ are transformed by translations and rotations to minimize the RMSD between the optimized backbone and the reference one. A similar library-based approach has been used in RNAfitme web-server for remodeling of nucleic-acid residue conformations of RNA structures [16].

Noteworthy, the original force field parameters were subject to minor tuning, to generate structures with better MolProbity scores. We changed the rest values of OP1-P-OP2 and N9-C1’-O4’ angles to 119.62° and 109.00° respectively, thereby allowing for the elimination of most ‘bad angles’ reported by MolProbity.

### Custom restraints

Distance restraints are implemented as simple harmonic forces, as given by Eq. (4).

4 $$ {E}_{spring}={k}_4{\left(\overrightarrow{r_i}-\overrightarrow{c_i}\right)}^2 $$

*k*_*4*_ denotes the force constant which can be set by the user. The spring forces can be used as positional or distance restraints since their anchor points *c*_*i*_ can be constituted by both atoms and arbitrary points in space.

### Minimization

After setting up the model, QRNAS starts to minimize the energy of the system. All force field terms in our model are analytically differentiable, enabling us to use minimization schemes with explicit gradient information. We implemented two algorithms: steepest descent with golden section search and Polak-Ribiere conjugate gradients [17].

### Performance optimization

Calculation of electrostatics was parallelized for machines with symmetric multiprocessing (SMP) capability, i.e., multicore workstations. Parallelism was achieved by processing of the ‘electrostatic interaction matrix’ in blocks that share no common atoms. Consequently, the proposed algorithm is nearly lock-free and has much-improved cache hit rate compared to a version which processes pairwise interactions in a random order. We tuned the parameters of the algorithm (block size and pointer hashing function) to achieve good performance on workstations with up to 8 cores. As a proof of concept, we successfully conducted minimization of ribosomal RNA taken from the 60S subunit of the eukaryotic ribosome (PDB code: 4A18) achieving the performance of 0.2 golden-section search steps per hour.

Example run-times for representative models of RNA structure analyzed in this paper, minimized for 1000 steps on a single core of 2.40 GHz Intel® Xeon-E5620 CPU (Linux 4.15.0–45-generic-x86_64/Ubuntu 18.04.1 with g++/gcc 7.3.0 compiler) with/without new options (explicit hydrogen bonds, base pair co-planarity, and backbone regularization): 1byx (16 residues): 39.48 s/39.12 s; 2lu0 (49 residues): 254.00 s /250.19 s; 2jyf (86 residues): 689.26.s /685.86 s.

## Results

### Regularization of NMR structures

First, we tested QRNAS on a set of twelve nucleic acid 3D structures determined by solution NMR (1A60 [18], 1B36 [19], 2L7D [20], 1P5M [21], 1YG3 [22], 2JYF, 2LC8 [23], 2 LU0 [24], 2M4Q [25], 2 M58 [26], 1BYX [27], 1DXN [28] in the Protein Data Bank). The common feature of the targets chosen for this analysis were suboptimal scores reported by MolProbity [9]. The test set included mostly RNA structures, except for three chimeric and hybrid (RNA/DNA) structures (2L7D, 1BYX, 1DXN). Whenever an ensemble of models was present, we used the first model. All models except two (2LC8, 1BYX) suffered from high clash-scores. All models except two (2L7D, 1DXN) were reported as having bad backbone conformations. Some bad bonds were detected in 1A60, 1YG3 and bad angles were found in 1A60, 1YG3, 2LC8, 2 M58, 1BYX, 1DXN respectively.

We used QRNAS with restraints on explicit hydrogen bonds, restraints on base pair co-planarity, and backbone regularization. No custom restraints were used at this stage. QRNAS was able to resolve all clashes in the studied set, outperforming both the RNAfitme web server (which uses NAMD with CHARMM force-field for optimizing RNA structures) and sander from the AMBER package (Table 1). The mean amount of bad angles was reduced from 3.46 to 1.31%. The average fraction of wrong backbone conformations was reduced from 27.43 to 14.83%. On the contrary, RNAfitme and sander increased the percentages of bad angle and wrong backbone conformations upon refinement. None of the methods has shown consistent improvement of the fraction of bad bonds. This analysis demonstrates the ability of QRNAS to regularize structures and improve their MolProbity scores, and also shows the limitations of current methods. For practical application of QRNAS to optimize NMR-derived RNA models it will be worthwhile to use NMR-derived data as additional custom restraints in the optimization process and to validate the optimized structures against the NMR data that were not used in the optimization.

**Table 1:** Performance of QRNAS on a selection of NMR structures in terms of optimization of MolProbity scores. QRNAS resolved nearly all steric clashes. It also improved backbone conformations and bond lengths in all studied cases at the price of small perturbations in the angle space. Quality scores of models optimized with RNAfitme and *sander* from the AMBER package are shown for comparison. In three cases, RNAfitme was unable to process the input file

PDB ID	Clashscore				Bad backbone conf. [%]				Bad bonds [%]				Bad angles [%]			
	Starting	QRNAS	RNAfitme	sander	starting	QRNAS	RNAfitme	sander	starting	QRNAS	RNAfitme	sander	starting	QRNAS	RNAfitme	sander
1A60	87.14	0.00	30.68	79.94	26.19	16.66	25.58	26.19	0.34	0.34	0.17	0.34	1.63	0.65	2.02	1.74
1B36	23.75	0.00	12.26	25.90	52.63	25.00	52.63	55.56	0.00	0.40	0.00	0.40	0.00	2.54	0.36	0.00
2L7D	14.47	0.00	–	14.99	0.00	0.00	–	0.00	0.00	0.00	–	1.52	0.00	0.00	–	0.00
1P5M	27.87	0.00	20.45	28.26	18.18	9.44	18.18	16.98	0.00	0.27	0.00	0.27	3.66	0.77	3.81	3.18
1YG3	85.46	0.00	30.94	91.22	64.29	53.85	64.29	69.23	2.58	0.55	2.56	3.29	4.44	4.40	4.58	4.75
2JYF	66.93	0.00	–	64.75	9.30	12.20	–	9.76	0.00	0.35	–	0.35	0.00	1.00	–	0.35
2LC8	0.00	0.00	0.55	0.00	12.50	5.56	12.50	12.96	0.00	0.26	0.00	0.26	22.79	0.08	22.78	22.71
2 LU0	64.09	0.00	23.45	64.43	44.90	25.53	44.90	46.81	0.00	0.30	0.00	0.30	0.00	1.55	0.28	0.00
2M4Q	23.09	0.00	16.13	21.38	7.41	0.00	7.41	8.00	0.00	0.57	0.00	0.57	0.00	0.73	4.07	4.21
2 M58	13.16	0.00	9.47	13.86	50.85	31.57	50.85	50.88	0.00	0.25	0.00	0.25	0.16	2.16	0.46	0.16
1BYX	0.00	0.00	–	0.00	41.66	0.00	–	33.33	0.00	1.68	–	1.20	2.13	0.74	–	2.40
1DXN	19.58	0.00	10.00	18.92	0.00	0.00	0.00	0.00	0.32	0.00	0.00	1.50	4.83	0.42	6.45	5.69

The first models from the NMR were used in this analysis. The PDBs that contains DNA/hybrid were not analyzed using RNAfitme and represented by ‘—‘in the table

### Assessment of model accuracy

In molecular modeling, one of the essential steps is the selection of the potentially best models. Once the different conformations are generated, a scoring function can be applied to assess the global and local features of the model, aiming at discriminating models that are closer to the ‘true’ structure (usually represented as a model obtained in the course of X-ray crystallography or NMR experiments and used as a reference) from those that are less accurate. While the selection of models was not the primary goal of QRNAS, we tested its ability to score models. In general, in our various analyses, we did not observe the correlation of QRNAS single point energy values (combined with additional scoring from our custom terms) with the model quality (data not shown) [6, 7, 29–31]. We suspected that this might be caused by the fine-grained character of the scoring function and its extreme sensitivity to the ruggedness of the RNA energy landscape. In other words, we expected that QRNAS might be able to discriminate ‘good’ and ‘bad’ models only very close to the global energy minimum corresponding to the reference structure. On the other hand, in typical modeling exercises, models generated computationally are relatively far from the reference structure, and their RMSD values rarely fall below 5 Å.

**Fig. 1:**
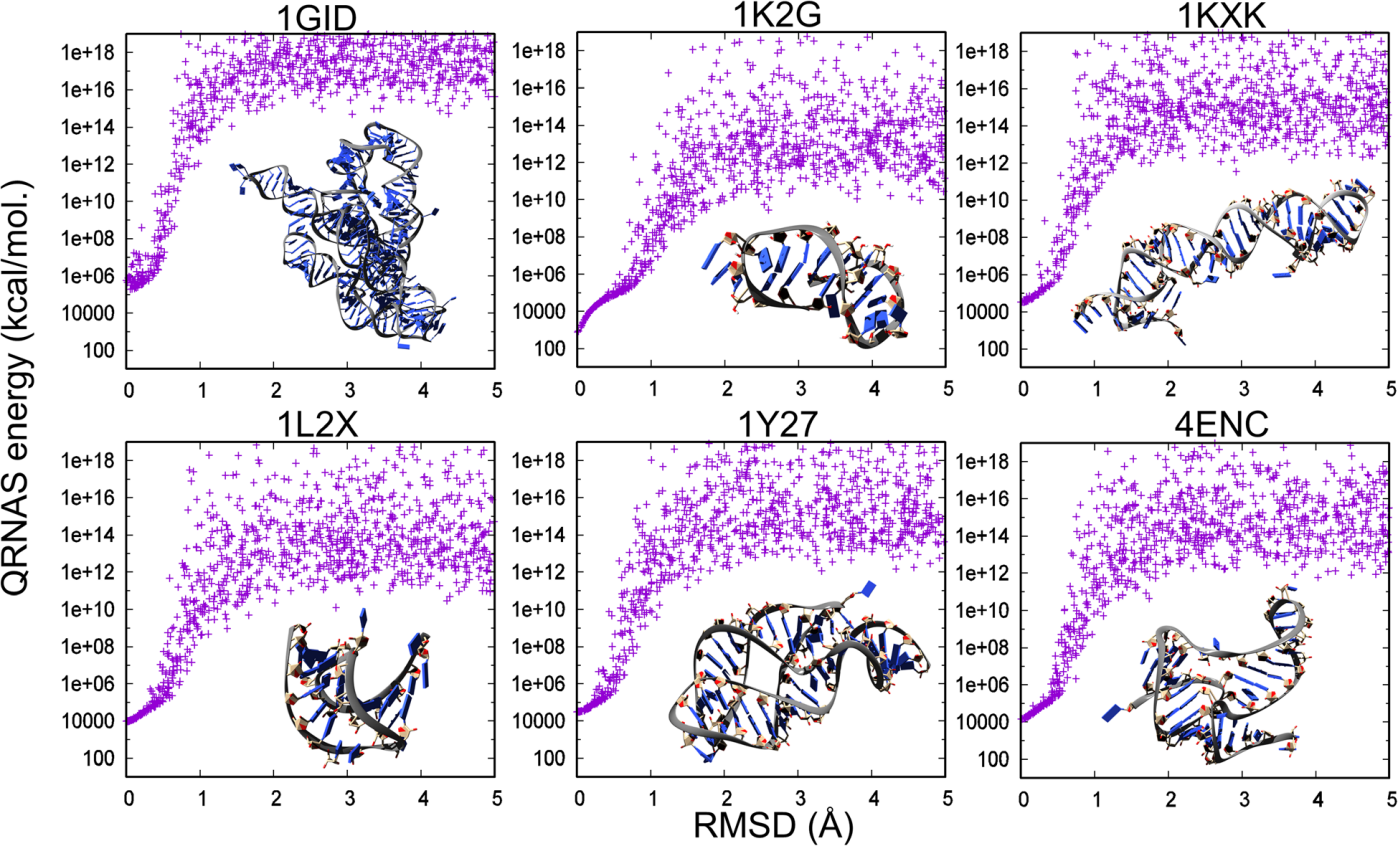
QRNAS single point energy vs. RMSD on sets of decoys derived from the six different experimentally determined structures (1GID, 1KXK, 1L2X, 1Y27, and 4ENC solved by X-ray crystallography and 1K2G by NMR). No correlation between the QRNAS score and model quality is observed, except for the immediate vicinity of the reference structures (RMSD 0–2 Å). 3D models of the native structures are displayed as an inset in the respective plots

Instead of looking at models generated by folding simulation, we started from six experimentally determined structures which include P4-P6 ribozyme domain of group I intron (PDB code: 1GID [32]), GBS/omegaG group-I intron (PDB code: 1K2G [33]), ai5-gamma group II self-splicing intron (PDB code: 1KXK [34]), viral RNA pseudoknot (PDB code: 1L2X [35]), G-riboswitch aptamer (PDB code: 1Y27 [36]), and fluoride riboswitch (PDB code: 4ENC [37]); and we generated models by introducing minor random perturbations to positions of all atoms. From the pool of generated models, we selected 1000 structures with RMSD to the starting/reference structure ranging from near 0.00 to 5.00 Å. Scoring these models with QRNAS revealed a funnel-like shape, indicative of an energy/score minimum near the native structure (Fig. 1). Alas, the funnel was very narrow, less than 2 Å, which indicated that QRNAS could discriminate only between models that were extremely close to the reference and all the others, but it was incapable of discriminating between models that are very good (RMSD, e.g., around 2 Å) and those that are much worse. This also suggested that the optimization of QRNAS score (e.g., in the course of model refinement) is unlikely to improve the global accuracy of models unless the starting models are already extremely close to the ‘true’ structure. For models of lower accuracy, statistical potentials can be used, such as RASP [38] or the energy functions used in 3D structure prediction methods such as SimRNA [31, 39] or ROSETTA/FARNA/FARFAR [40, 41]. It is worth emphasizing that computational improvement of model accuracy remains a difficult problem, for which no perfect solution exists. QRNAS addresses one of the aspects of this problem, at the level of local geometry.

### Refinement of models in RNA-puzzles experiment

We analyzed the performance of QRNAS on models for two targets of the RNA-Puzzles experiment (Puzzle #1 – relatively easy [5], Puzzle #6 – very difficult [6]), and the resulting broad range of model accuracy. We analyzed up to five top first structures submitted by various participants, generated with different modeling methods, and hence presenting different types of errors and inaccuracies. The modeling methods used by different groups for Puzzles #1 and #6 include ModeRNA [42] and SimRNA [31, 39] (Bujnicki group), Vfold [43] (Chen group), FARNA/FARFAR [40, 41] (Das group), iFoldRNA [44] (Dokholyan group), MC-Fold|MC-Sym [45] (Major group), and RNA123 software suite [46] (SantaLucia group). The models were obtained from the RNA-Puzzles experiment website (currently: http://rnapuzzles.org/). In Puzzle #1 the average RMSD of models was 4.93 Å (best model exhibited 3.42 Å), while in Puzzle #6 the model deviated from the reference structure by 23.05 Å on the average (best model exhibited 11.29 Å).

To assess the capabilities of QRNAS, we conducted a full refinement with default parameters for 10,000 steps. For comparison, we performed refinement with RNAfitme and minimization with sander from the Amber 14 package [47]. RNAfitme was run with the default settings on the web server. Minimization with sander was performed in a truncated octahedral box of 10 Å with TIP3P water model [48] and leaprc.ff14SB variant of the forcefield [49, 50]. The following parameters were used while running sander: imin 1, maxcyc 10,000, cut 300, igb 2, saltcon 0.2, gbsa 1, ntpr 10, ntx 1, ntb 0. For the resulting models, we calculated the value of global RMSD to assess the overall accuracy, and the Interaction Network Fidelity (INF) to compare the accuracy of residue-residue contacts identified in the original and optimized structures [51]. INF values are calculated for all types of contacts including canonical and non-canonical base-pairs and stacking. For the detection of base pairs, we have used our in-house method ClaRNA [52].

In all cases, QRNAS improved MolProbity scores, in particular, it resolved nearly all steric clashes (Tables 2 and 3). For Puzzle #1 (Table 2), the average change of RMSD was − 0.01 for QRNAS vs. 0.26 for sander (i.e., essentially no change vs. minimal deterioration). However, the average INF value decreases from 0.802 to 0.768, 0.759, and 0.482, calculated from the optimized models using QRNAS, sander and RNAfitme web server, respectively. For Puzzle #6 (Table 3) the average change of RMSD was 0.53 for QRNAS vs. 0.51 for sander and 0.52 for RNAfitme (negligible deterioration), and the average improvement of INF was 0.001 (for QRNAS) compare to 0.00 (for sander) and − 0.04 (for RNAfitme) in respect to the starting models. To evaluate the performance of QRNAS to see how it can optimize the non-canonical contacts, we have calculated INF considering only the non-Watson-Crick contacts (INF_nWC) for the models of RNA-Puzzles #1 and #6. In both the rounds, QRNAS improved the INF_nWC values with respect to the starting models. Though QRNAS and RNAfitme have comparable (very minor) improvement of non-canonical contacts, sander does not improve such contacts. Summarizing, in terms of RMSD, the structures changed very little; sometimes the models improved slightly, sometimes they deteriorated slightly. This was expectable because in all cases the models were so far from the reference structure that the local refinement was not expected to drive them towards the global energy minimum, but rather towards a local minimum, which could be further away from the reference structure. On the other hand, we could observe a small increase in the INF values, indicating a small improvement of predicted contacts. We attribute this small change to the ability of QRNAS to improve the local geometry, in particular in the case of base pairs. In models that are reasonably close to the ‘true’ structure and exhibit residues that are ‘almost’ in proper contact with each other (as in many models for Puzzle #1), the optimization by QRNAS can refine these contacts and enable the formation of proper base pairs. The smaller improvement of contacts in models of Puzzle #6 can be explained by the low quality of the starting structures, and the lower fraction of ‘nearly correct’ contacts that could be optimized.

**Table 2:** Performance of QRNAS on RNA Puzzle #1 models in terms of model accuracy, as compared to RNAfitme and sander from the AMBER package

RNA-Puzzles #1 models	RMSD [Å]				INF_all				INF_nWC				Clashscore			
	starting	QRNAS	RNAfitme	sander	starting	QRNAS	RNAfitme	sander	starting	QRNAS	RNAfitme	sander	starting	QRNAS	RNAfitme	sander
1_bujnicki_1	5.70	5.63	5.60	5.63	0.825	0.778	0.522	0.787	0.750	0.750	0.866	0.750	0.00	0.00	0.00	0.00
1_bujnicki_2	6.13	5.98	5.93	5.97	0.776	0.774	0.496	0.736	0.289	0.671	0.500	0.289	61.25	1.40	18.91	60.88
1_bujnicki_3	5.28	5.24	5.22	5.23	0.796	0.761	0.483	0.750	0.707	0.408	0.866	0.707	48.21	2.10	10.81	48.32
1_bujnicki_4	4.94	4.87	4.84	4.84	0.703	0.654	0.357	0.652	0.250	0.378	0.354	0.250	69.06	0.70	14.86	70.22
1_bujnicki_5	5.11	4.91	4.95	5.01	0.701	0.664	0.376	0.656	0.378	0.333	0.378	0.378	66.30	2.10	23.65	68.12
1_chen_1	4.34	4.32	4.31	4.38	0.901	0.809	0.457	0.789	0.000	0.577	0.289	0.000	0.00	0.00	0.00	0.00
1_das_1	3.96	4.06	4.08	4.06	0.812	0.812	0.499	0.812	0.671	0.671	0.577	0.671	2.80	0.00	0.00	2.80
1_das_2	4.46	4.46	4.48	4.45	0.778	0.769	0.499	0.778	0.500	0.671	0.750	0.500	3.50	0.00	0.00	4.20
1_das_3	3.42	3.48	3.50	3.48	0.843	0.851	0.522	0.843	0.894	0.894	0.750	0.894	2.80	0.00	0.00	4.20
1_das_4	3.91	4.12	4.12	4.11	0.819	0.818	0.522	0.819	0.816	0.816	0.816	0.816	2.80	0.00	0.00	2.80
1_das_5	4.56	4.80	4.79	4.79	0.743	0.750	0.480	0.743	0.750	0.577	0.577	0.750	2.80	0.00	0.00	2.80
1_dokholyan_1	7.18	7.12	7.10	7.13	0.785	0.720	0.503	0.737	0.671	0.500	0.500	0.671	26.15	0.00	10.13	26.59
1_major_1	4.32	4.47	4.48	4.43	0.885	0.814	0.522	0.770	0.577	0.866	0.866	0.577	54.99	2.78	14.19	51.89
1_santalucia_1	5.75	5.46	5.41	5.43	0.864	0.776	0.510	0.755	0.577	0.577	0.577	0.577	25.07	0.00	8.78	21.71
average	4.93	4.92	4.92	4.92	0.802	0.768	0.482	0.759	0.559	0.621	0.619	0.559	26.12	0.65	7.24	26.04

**Table 3:** Performance of QRNAS on RNA Puzzle #6 models in terms of model accuracy, as compared to RNAfitme and sander from the AMBER package

RNA Puzzle #6 model	RMSD [Å]				INF_all				INF_nWC				Clashscore			
	Starting	QRNAS	RNAfitme	sander	Starting	QRNAS	RNAfitme	sander	Starting	QRNAS	RNAfitme	sander	Starting	QRNAS	RNAfitme	sander
6_blanchet_1	21.39	22.29	22.34	22.30	0.761	0.754	0.742	0.761	0.462	0.462	0.405	0.462	0.74	0.00	1.28	0.55
6_blanchet_2	20.94	21.76	21.77	21.75	0.744	0.726	0.713	0.744	0.434	0.418	0.316	0.434	0.37	0.00	0.18	0.37
6_blanchet_3	20.57	21.32	21.32	21.31	0.745	0.754	0.696	0.745	0.405	0.452	0.337	0.405	0.55	0.00	1.10	0.74
6_blanchet_4	21.46	22.21	22.24	22.22	0.763	0.752	0.724	0.763	0.418	0.434	0.372	0.418	0.55	0.18	1.47	0.55
6_blanchet_5	23.54	24.19	24.18	24.18	0.724	0.728	0.706	0.724	0.337	0.372	0.337	0.337	0.74	0.55	0.92	0.74
6_bujnicki_1	36.50	37.00	36.98	36.96	0.720	0.721	0.729	0.720	0.300	0.224	0.194	0.300	0.55	0.00	0.55	0.55
6_bujnicki_2	30.47	30.93	30.90	30.89	0.701	0.692	0.699	0.701	0.254	0.337	0.323	0.254	1.29	0.18	1.10	1.29
6_bujnicki_3	31.79	32.14	32.11	32.09	0.637	0.638	0.651	0.637	0.258	0.258	0.270	0.258	1.11	0.37	0.92	1.11
6_bujnicki_4	31.64	32.07	32.05	32.04	0.657	0.652	0.646	0.657	0.135	0.135	0.115	0.135	1.84	0.18	1.28	1.84
6_chen_1	23.89	24.30	24.29	24.29	0.673	0.676	0.690	0.673	0.200	0.183	0.237	0.200	0.37	0.00	0.37	0.37
6_chen_2	21.73	22.13	22.17	22.15	0.656	0.662	0.663	0.656	0.200	0.283	0.254	0.200	0.18	0.18	0.55	0.18
6_chen_3	23.25	23.62	23.62	23.63	0.681	0.674	0.685	0.681	0.200	0.200	0.183	0.200	0.55	0.18	0.55	0.55
6_chen_4	21.71	22.15	22.11	22.12	0.669	0.688	0.699	0.669	0.224	0.298	0.316	0.224	1.84	0.18	1.65	1.84
6_chen_5	23.17	23.56	23.52	23.53	0.672	0.676	0.676	0.669	0.224	0.149	0.183	0.224	17.51	10.69	12.29	17.14
6_das_2	13.05	13.48	13.46	13.45	0.765	0.766	0.744	0.765	0.422	0.462	0.488	0.422	20.65	0.00	10.64	20.28
6_das_3	15.26	15.57	15.54	15.54	0.756	0.755	0.750	0.756	0.488	0.513	0.422	0.488	18.79	0.18	7.89	19.90
6_das_4	11.29	11.62	11.61	11.59	0.766	0.770	0.749	0.766	0.488	0.513	0.537	0.488	28.02	4.98	15.04	28.39
6_das_5	15.29	15.58	15.57	15.56	0.782	0.796	0.789	0.782	0.488	0.537	0.474	0.488	14.74	0.00	6.97	14.56
6_dokholyan_1	25.32	26.07	26.05	26.05	0.705	0.705	0.704	0.705	0.323	0.299	0.488	0.323	11.06	0.00	6.97	11.24
6_dokholyan_2	25.92	26.58	26.57	26.55	0.703	0.718	0.706	0.703	0.298	0.270	0.239	0.298	9.40	0.00	5.50	9.22
6_dokholyan_3	25.58	26.21	26.20	26.18	0.691	0.696	0.689	0.691	0.298	0.298	0.283	0.298	9.22	0.00	5.69	9.40
6_dokholyan_4	24.27	24.95	24.95	24.93	0.708	0.691	0.725	0.708	0.338	0.338	0.299	0.338	9.59	0.00	6.42	9.95
6_dokholyan_5	22.07	22.62	22.60	22.58	0.704	0.708	0.709	0.704	0.338	0.316	0.447	0.338	10.51	0.00	7.71	10.69
average	23.05	23.58	23.57	23.56	0.712	0.713	0.708	0.712	0.327	0.337	0.365	0.327	6.96	0.78	4.22	7.02

### Previously published examples of QRNAS application

Following the development and initial tests of QRNAS, we applied it in various modeling studies. In the course of collaborative work on models generated by all groups for Puzzles #5, #6, and #10, we found that models submitted by the Das group had poor clash scores, despite their overall relative accuracy, as measured in terms of RMSD to the reference structure. We have therefore run QRNAS on all Das models submitted for Puzzles #5, #6, and #10 (17 models total). In all cases, a dramatic reduction of clash scores was obtained; in 10 models even down to zero. Only in three cases, the clash scores remained larger than 4; however, these models had initial Clash Scores of nearly 30. Details of this analysis were reported in an article describing RNA-Puzzles Round II [6].

In order to evaluate the performance of QRNAS for blind predictions (at the time when the experimentally determined structure was not available), we calculated the MolProbity scores of RNA-Puzzles #6 models generated in our group before the refinement. The MolProbity scores show improvement in the quality of the models as the average Clashscores reduced from 8.99 to 1.99 (Table 4). The current version of QRNAS has also reduced the bad conformations, bad angles, and bad bonds in the models submitted for RNA-Puzzles #6 (Table 3).

**Table 4:** Performance of QRNAS for RNAs with unknown reference structures. MolProbity scores of “before” and “after” QRNA optimizations of the models generated in the Bujnicki group for RNA-Puzzles # 6

Models	Clashscores		Bad conformations [%]		Bad bonds [%]		Bad angles [%]	
	Before	After	Before	After	Before	After	Before	After
6_Bujnicki_1	4.95	0.55	25.00	11.31	0.07	0.00	2.35	0.52
6_Bujnicki_2	8.99	1.28	23.81	14.88	0.02	0.00	2.86	0.63
6_Bujnicki_3	9.36	1.10	25.60	15.48	0.20	0.00	3.52	0.51
6_Bujnicki_4	12.66	1.83	26.19	17.86	0.22	0.00	4.25	0.49
Average	8.99	1.19	25.15	14.88	0.13	0.00	3.25	0.54

In the case of group I intron modeling study [29], QRNAS was used as the final step of a workflow to improve a model generated with ModeRNA [42] and SimRNA [31]. It reduced the clash-score from 184.69 to 0.37, bad bonds from 4.12 to 0.00%, bad angles from 6.53 to 0.88%, without major changes of the deviation from the reference structure (10.9 Å to 11.0 Å).

## Conclusions

QRNAS is a software tool for fine-grained refinement of nucleic acid structures, based on the AMBER force field with additional restraints. QRNAS is capable of handling RNA, DNA, chimeras, and hybrids thereof, and enables modeling of nucleic acids containing modified residues. We demonstrate the ability of QRNAS to improve the quality of RNA 3D structure models generated with different methods. QRNAS was able to improve MolProbity scores of NMR structures, as well as of computational models generated in the course of the RNA-Puzzles experiment. The overall geometry improvement may be associated with the improvement of local contacts, but the systematic improvement of root mean square deviation to the reference structure should not be expected. QRNAS can be integrated into a computational modeling workflow with other tools, enabling improved RNA 3D structure prediction. Our group systematically uses QRNAS at the final stage of model refinement in the context of the RNA-Puzzles experiment.

## Availability and requirements

Project name: QRNAS

Project home page: http://genesilico.pl/software/stand-alone/qrnas

GitHub page (Mirror): https://github.com/sunandanmukherjee/QRNAS.git

Operating systems: GNU/Linux, MacOS and WSL on Windows 10.

Programming language: C++

License: GNU GPLv3+

Any restrictions to use by non-academics: None

For the compilation of QRNAS, a C++ compiler, such as GNU g++ is required. A Makefile is provided for the compilation of the package. Download the software from http://genesilico.pl/software/stand-alone/qrnas or clone it from https://github.com/sunandanmukherjee/QRNAS.git. Unzip the archive, and compile it with the command make to create an executable version of QRNAS. To execute the program use the command …/path/to/QRNAS/QRNA –i input.pdb –o output.pdb where input.pdb is the file to be optimized and output.pdb is the optimized structure. For more advanced usage of QRNAS, users should consult the user manual and the README.txt file in the QRNAS package.

## Abbreviations

INF: Interaction Network Fidelity
PDB: Protein Data Bank
RMSD: Root mean square deviation
